# Three-Year Outcomes of iStent Inject W Implantation Combined With Phacoemulsification in Japanese Eyes With Open-Angle Glaucoma: A Retrospective Study

**DOI:** 10.7759/cureus.94885

**Published:** 2025-10-18

**Authors:** Takahiro Usami, Takahiro Matsuoka, Masami Yamashita, Ryosuke Yamada, Ayano Tanaka, Hiroki Kaneko

**Affiliations:** 1 Department of Ophthalmology, Hamamatsu University School of Medicine, Hamamatsu, JPN; 2 Department of Ophthalmology, Chutoen General Medical Center, Kakegawa, JPN; 3 Department of Ophthalmology, Shizuoka Red Cross Hospital, Shizuoka, JPN

**Keywords:** intraocular pressure, istent inject w, japanese population, minimally invasive glaucoma surgery (migs), normal-tention glaucoma, primary open-angle glaucoma (poag)

## Abstract

Background

Glaucoma is a leading cause of irreversible blindness. In Japan, most primary open-angle glaucoma (POAG) cases are normal-tension glaucoma (NTG). Micro-invasive glaucoma surgery (MIGS) such as the iStent inject W (Glaukos Corporation, San Clemente, CA) offers a less invasive option, but long-term outcomes in NTG-predominant populations are scarce.

Objective

The objective of this study is to evaluate three-year outcomes of combined phacoemulsification and iStent inject W implantation in Japanese eyes with POAG.

Methods

This retrospective study included 24 eyes from 14 Japanese patients undergoing combined surgery between February 2021 and May 2022 at Chutoen General Medical Center. Eyes with prior glaucoma surgery (except laser trabeculoplasty) or secondary glaucoma were excluded. Intraocular pressure (IOP) and medication scores were recorded up to 36 months postoperatively. Changes from baseline were analyzed with the Wilcoxon signed-rank test.

Results

Mean IOP decreased from 16.71 ± 4.54 mmHg to 12.63 ± 2.59 mmHg at 36 months (p < 0.001), 24% reduction. Medication scores decreased from 3.17 ± 1.55 to 1.42 ± 1.10 (p < 0.001). At 36 months, out of 24 eyes, eight eyes (33.3%) were medication-free, and 14 eyes (58.3%) achieved ≥20% IOP reduction. No additional glaucoma surgeries were required. Complications were minimal, with transient hyphema in two eyes (8.3%) and transient IOP elevation at one week in two eyes (8.3%).

Conclusion

iStent inject W combined with phacoemulsification provided safe and sustained IOP and medication reduction over three years in Japanese eyes with POAG, supporting its use in NTG-predominant populations.

## Introduction

Glaucoma is a leading cause of irreversible blindness worldwide, with more than 70 million affected individuals, and its prevalence is projected to rise substantially as populations age [1,2]. In Japan, the Tajimi Study revealed that the majority of primary open-angle glaucoma (POAG) cases are normal-tension glaucoma (NTG), highlighting an epidemiological profile distinct from Western countries [3].

Randomized controlled trials, including the Early Manifest Glaucoma Trial, the Ocular Hypertension Treatment Study, and the Collaborative Normal-Tension Glaucoma Study, have established that lowering intraocular pressure (IOP) reduces the risk of disease progression, even in NTG patients [4-7]. However, achieving meaningful IOP reduction in NTG often requires long-term topical therapy or filtering surgery. Medical therapy is limited by adherence issues, ocular surface disease, and systemic side effects, while filtering surgery carries risks of hypotony, infection, and vision-threatening complications [8].

Micro-invasive glaucoma surgery (MIGS) has emerged as a less invasive option to improve aqueous outflow while maintaining a favorable safety profile [9]. Among MIGS devices, the iStent inject W (Glaukos Corporation, San Clemente, CA) places two trabecular micro-bypass stents into Schlemm’s canal to enhance conventional outflow. Previous studies have reported promising results, with sustained IOP and medication reduction after implantation combined with phacoemulsification [10-12]. However, many reports come from Western populations with predominantly high-tension glaucoma, and evidence in Asian populations, where NTG predominates, remains limited.

Therefore, the purpose of this study was to evaluate the three-year outcomes of combined phacoemulsification and iStent inject W implantation in Japanese eyes with POAG. We aimed to assess long-term changes in IOP, medication burden, and safety profile in a cohort characterized by advanced disease and a high prevalence of NTG.

## Materials and methods

Study design and participants

This retrospective observational study was conducted at Chutoen General Medical Center, Shizuoka, Japan. Consecutive patients who underwent combined iStent inject W implantation and phacoemulsification with intraocular lens implantation between February 2021 and May 2022 were screened. Inclusion criteria were (1) diagnosis of open-angle glaucoma (including POAG and NTG) and (2) follow-up of at least 36 months after surgery at our institution. Eyes with prior glaucoma surgery other than laser trabeculoplasty, secondary glaucoma, or incomplete follow-up data were excluded.

Diagnostic definitions

Diagnostic definitions followed the Japan Glaucoma Society Fifth Edition guidelines [9]. POAG was defined as open anterior chamber angles without secondary causes, together with glaucomatous optic neuropathy and corresponding visual-field loss on standard automated perimetry, accompanied by a documented, untreated IOP >21 mmHg at diagnosis or in historical records. NTG was defined by the same structural and functional criteria as untreated IOP, consistently ≤21 mmHg on repeated measurements (preferably including diurnal testing). For analytic consistency, eyes without a documented untreated IOP profile were conservatively classified as POAG.

Data collection and outcome measures

Baseline demographic and clinical data, including age, sex, glaucoma type, axial length, and baseline visual field mean deviation (VFMD), were obtained from medical records. The primary outcomes were changes in IOP, glaucoma medication score, and VFMD from baseline to each follow-up time point. IOP and medication score were recorded at baseline (preoperative) and at one day, one week, one, three, six, 12, 18, 24, 30, and 36 months postoperatively. VFMD was recorded at baseline (preoperative) and 12, 24, and 36 months postoperatively. The medication score was calculated by assigning one point per single-agent medication and two points for a fixed-combination drug. Medication tapering and reintroduction were at the surgeon's discretion based on individualized target IOP, ocular surface tolerance, and postoperative course.

Measurements

IOP was measured using Goldmann applanation tonometry (GAT) (Haag-Streit AG, Köniz, Switzerland). When reliable GAT was not feasible due to strong eyelid squeezing/blepharospasm, rebound tonometry (iCare, Icare Finland Oy, Vantaa, Finland) was used. For each eye, the same measurement modality (GAT or iCare) was maintained across all follow-up visits. No correction for central corneal thickness was applied. Readings were obtained during routine clinic hours, and the exact time of day was not standardized, which may introduce diurnal variation, particularly in NTG-predominant cohorts.

Visual fields were assessed with Humphrey Field Analyzer 3 (HFA3; Carl Zeiss Meditec, Jena, Germany) using the 30-2 program and SITA-Standard strategy. Only examinations deemed reliable per institutional criteria were included; VFMD was taken as the instrument-reported mean deviation.

Axial length was measured with IOLMaster 700 (Carl Zeiss Meditec, Jena, Germany) under dilated conditions; values were recorded according to the device’s automated averaging protocol.

Surgical procedure

All surgeries were performed by experienced glaucoma surgeons under topical anesthesia. Phacoemulsification (through a 2.4-mm temporal clear corneal incision) and IOL implantation were carried out first, followed by insertion of two iStent inject W devices into the nasal trabecular meshwork under gonioscopic view.

Safety definitions (intraoperative adverse events)

For this study, intraoperative adverse events were predefined as follows.

Stent-related: (1) inability to implant both iStent inject W devices as planned, (2) stent dislocation, (3) device occlusion by tissue or blood, and (4) iris injury attributable to device insertion.

Cataract-related: (1) posterior capsule rupture, (2) zonular weakness or dialysis, and (3) vitreous prolapse/loss.

Statistical analysis

Continuous variables are summarized as mean ± standard deviation (SD), and changes are presented as mean differences with 95% confidence intervals (CIs) for interpretability. Statistical significance was tested using the Wilcoxon signed-rank test. The effect size r was calculated as Z/√N (paired observations). Multiplicity arising from repeated time-point comparisons was treated as exploratory and unadjusted. Proportions are reported with Wilson 95% CIs. A two-sided p < 0.05 was considered statistically significant. Analyses were conducted using standard statistical software.

Ethical approval

This study was approved by the Institutional Review Board of Chutoen General Medical Center, Shizuoka, Japan (approval no. 1306250512). The study adhered to the tenets of the Declaration of Helsinki. Given the retrospective design, written informed consent was waived, and an opt-out process was adopted through institutional website disclosure.

## Results

Patient characteristics

A total of 24 eyes from 14 patients (eight men, six women) were included in the analysis. The mean age was 67.6 ± 9.7 years. All patients were Japanese. Out of 24 eyes, 13 eyes (54.2%) had NTG, and 11 eyes (45.8%) had POAG. The mean baseline VFMD was -11.82 ± 7.59 dB, and the mean axial length was 25.56 ± 1.30 mm (Table 1).

**Table 1:** Characteristics NTG: normal-tension glaucoma; POAG: primary open-angle glaucoma; VFMD: visual field mean deviation.

Characteristics	Value
Age (years), mean ± SD	67.6 ± 9.7
Gender, n (%)	
Male	8 (57.1%)
Female	6 (42.9%)
Race, n (%)	
Japanese	14 (100%)
Laterality, n (%)	
OD	13 (54.2%)
OS	11 (45.8%)
Type of glaucoma, n (%)	
POAG	11 (45.8%)
NTG	13 (54.2%)
VFMD (dB), mean ± SD	-11.82 ± 7.59
Axial length (mm), mean ± SD	25.56 ± 1.30

Intraoperative safety

No intraoperative adverse events occurred among the predefined categories: there were no instances of inability to implant both stents, stent dislocation, device occlusion by tissue/blood, or iris injury, and no posterior capsule rupture, zonular weakness/dialysis, or vitreous prolapse/loss.

Intraocular pressure

**Figure 1:**
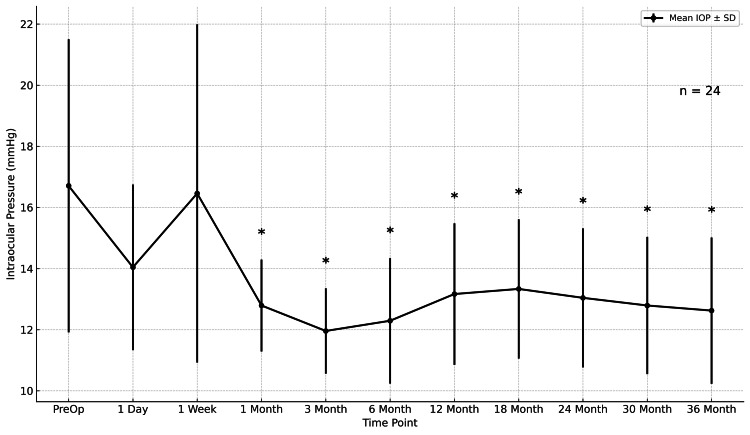
Mean IOP Mean IOP over 36 months. Values are mean ± 95% CI; n = 24. *p < 0.05 vs baseline (Wilcoxon signed-rank). IOP = intraocular pressure; CI = confidence interval.

Mean IOP decreased from 16.71 ± 4.54 mmHg preoperatively to 12.63 ± 2.59 mmHg at 36 months (p < 0.001), representing a mean reduction of -4.08 mmHg (95% CI, -5.76 to -2.40). The IOP reduction was statistically significant at all postoperative time points compared with baseline (Figure 1).

Medication score

**Figure 2:**
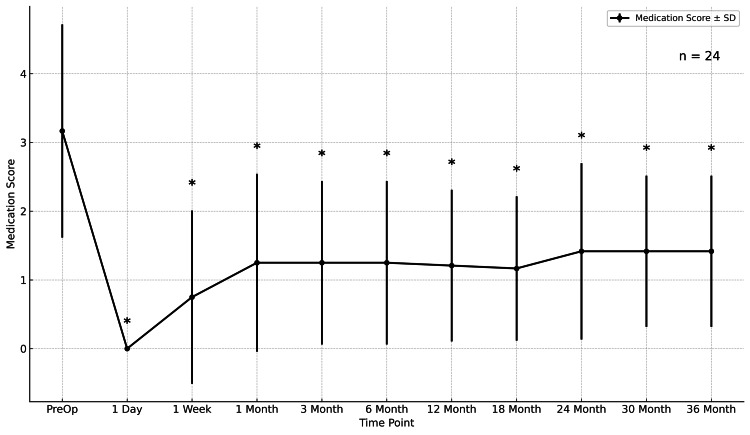
Medication score Mean medication score over 36 months. Values are mean ± 95% CI; n = 24. Medication score assigns one point per single-agent medication and two points per fixed-combination medication. *p < 0.05 vs baseline (Wilcoxon signed-rank). CI = confidence interval.

The mean glaucoma medication score decreased from 3.17 ± 1.55 preoperatively to 1.42 ± 1.10 at 36 months (p < 0.001), corresponding to a mean reduction of -1.75 (95% CI, -2.15 to -1.35). Significant reductions were observed at all follow-up visits (Figure 2).

Visual field mean deviation

VFMD remained generally stable through 36 months. Mean ± SD VFMD was -11.82 ± 7.76 dB preoperatively (n = 24), -11.74 ± 8.56 dB at 12 months (n = 20), -12.23 ± 6.97 dB at 24 months (n = 15), and -11.58 ± 8.75 dB at 36 months (n = 22). In paired analyses vs baseline, the median change was +0.72 dB at 12 months (n = 20; Wilcoxon p = 0.2455), +0.08 dB at 24 months (n = 15; p = 0.9341), and +0.09 dB at 36 months (n = 22; p = 0.4826).

Main outcomes at 36 months

At the final follow-up, out of 24 eyes, eight eyes (33.3%) were medication-free, 14 eyes (58.3%) achieved ≥20% IOP reduction from baseline, and only one eye (4.2%) experienced an increase in IOP compared to preoperative levels. No eyes required additional glaucoma surgery during the follow-up period (Table 2).

**Table 2:** Main outcomes VFMD analyses were performed on 22 eyes with both preoperative and 36-month data available. IOP: intraocular pressure; VFMD: visual field mean deviation.

Outcome	Preoperative	36 Months	Change (95% CI)	p-value	Effect size	Notes
IOP (mmHg), mean ± SD	16.71 ± 4.54	12.63 ± 2.59	-4.08 (-5.76 to -2.40)	<0.001	r = 0.986	Wilcoxon signed-rank
Medication score, mean ± SD	3.17 ± 1.55	1.42 ± 1.10	-1.75 (-2.15 to -1.35)	<0.001	r = 0.967	Wilcoxon signed-rank
VFMD (dB), mean ± SD	-11.82 ± 7.76	-11.58 ± 8.75	-0.81 (-2.21 to 0.59)	0.483	r = 0.15	Wilcoxon signed-rank（paired n = 22）
Medication-free eyes, n (%)	-	8 (33.3%)	-	-	-	8/24 eyes
Eyes with ≥20% IOP reduction, n (%)	-	14 (58.3%)	-	-	-	14/24 eyes
Eyes with IOP increase, n (%)	-	1 (4.2%)	-	-	-	Vs preoperative
Additional glaucoma surgery, n (%)	-	-	-	-	-	None

Postoperative complications

No eyes required reoperation for glaucoma during the 36-month follow-up. Postoperative hyphema with niveau formation was observed in two (8.3%) out of 24 eyes. Elevated IOP ≥30 mmHg occurred in 0 eyes (0.0%) on postoperative day 1, in two eyes (8.3%) at postoperative week 1, and in 0 eyes (0.0%) at postoperative month 1.

## Discussion

In the present study, combined phacoemulsification and iStent inject W implantation resulted in significant and sustained reductions in both IOP and medication burden over a 36-month follow-up period. Mean IOP decreased by over 4 mmHg (-24%) from baseline, and nearly 60% of eyes achieved ≥20% reduction. Furthermore, 13 eyes were medication-free at three years, despite a high baseline medication score of 3.2. No eyes required additional glaucoma surgery, underscoring the safety and durability of the procedure.

A notable observation at 36 months was that one eye (4.2%) showed a higher IOP than baseline, with an absolute change of +1 mmHg (14 to 15 mmHg), while its medication score decreased from 4 to 1. This pattern suggests that the modest IOP rise was more likely attributable to de-escalation of topical therapy rather than surgical failure. The increase was clinically mild and manageable by re-introducing or adjusting medications; no additional glaucoma surgery was required. Potential mechanisms include peri-stent fibrosis or stent obstruction [13] and segmental variability of distal outflow involving collector channels [14], as well as non-IOP-dependent processes relevant to NTG pathophysiology [15].

Our results compare favorably with previous reports. Salimi et al. demonstrated a 22% IOP reduction at 36 months in a single-center cohort from Canada [10]. Clement et al. reported significant short-term efficacy at 12 months in Australian patients [11]. A prospective seven-year cohort of iStent inject further supports long-term durability, reporting approximately 34-44% IOP and 58-76% medication reductions, no filtering surgeries over seven years, and clinically significant VFMD progression (≥2.5 dB) in 4.84%, albeit in a higher-IOP, predominantly non-Hispanic White population [16]. Importantly, Ang et al. recently reported outcomes specifically in Asian patients with NTG, showing modest IOP reduction but a marked decrease in medication burden, with over 80% of eyes medication-free at 12 months [12]. Despite including eyes with more advanced glaucoma (mean VFMD -11.8 dB in our study vs approximately -5 to -6 dB in other reports), our outcomes were comparable or better in terms of both IOP and medication reduction. Taken together with the findings of Ang et al. [12], our study reinforces the growing evidence that iStent inject W can provide meaningful long-term benefit even in NTG-predominant Asian populations, underscoring its clinical value in Japan, where NTG is the most common form of open-angle glaucoma.

The strengths of this study include its relatively long follow-up period, detailed outcome measures, and the focus on a Japanese cohort with advanced glaucoma and NTG predominance. These factors provide new insights into the applicability of iStent inject W in Asian populations. However, limitations include the retrospective, small, uncontrolled design, which limits generalizability. Because no standardized protocol governed postoperative medication tapering/re-initiation, decisions were made at the surgeon’s discretion, introducing management heterogeneity. Given the small cohort and few failure events, baseline-IOP-stratified analyses, modeling of within-patient correlation (when both eyes were included), and Kaplan-Meier survival analysis were not performed or would have been underpowered. Untreated IOP was unavailable for all eyes, so misclassification at the NTG/POAG boundary cannot be excluded. Larger multicenter prospective studies are needed to confirm long-term effectiveness in NTG-dominant populations.

## Conclusions

In conclusion, this study demonstrates that combined phacoemulsification and iStent inject W implantation provides sustained reductions in IOP and medication burden over three years in Japanese eyes with open-angle glaucoma. Despite including more advanced cases with higher medication requirements than most prior reports, outcomes were favorable and comparable to international studies. These findings suggest that MIGS can be a safe and effective treatment option even in populations characterized by advanced disease and a high prevalence of NTG.
